# Supplementary material for: The biogeochemical transport by the Gulf Stream
Source: Commun Earth Environ. 2026 Feb 12;7(1):149. doi: 10.1038/s43247-025-03118-y (PMC12900640; doi:10.1038/s43247-025-03118-y)
Supplement: Supplementary file 2 — Supplementary material [file 43247_2025_3118_MOESM2_ESM.pdf]

The biogeochemical transport by the Gulf Stream

Richard G. Williams [1,*], Peter J. Brown [2], Yohei Takano [3], Gaël Forget [4], Dani Jones [5], Anna Katavouta [6], Elaine McDonagh [7,2], and Vassil Roussenov [1]

[1]School of Environmental Sciences, University of Liverpool, Liverpool, L69 3GP, UK
[2]National Oceanography Centre, Southampton, UK
[3]British Antarctic Survey, Cambridge, UK
[4]Massachusetts Institute of Technology, Cambridge, USA
[5]University of Michigan, USA
[6]National Oceanography Centre, Liverpool, UK
[7]NORCE, Bergen, Norway
[*]ric@liverpool.ac.uk

Supplementary information

References

1. Williams, R. G. *et al.* Nutrient streams in the North Atlantic: Advective pathways of inorganic and dissolved organic nutrients. *Glob. Biogeochem. Cycles* **25** (2011).
2. Williams, R. G. & Follows, M. J. *Ocean Dynamics and the Carbon Cycle: Principles and Mechanisms* (Cambridge University Press, 2011).

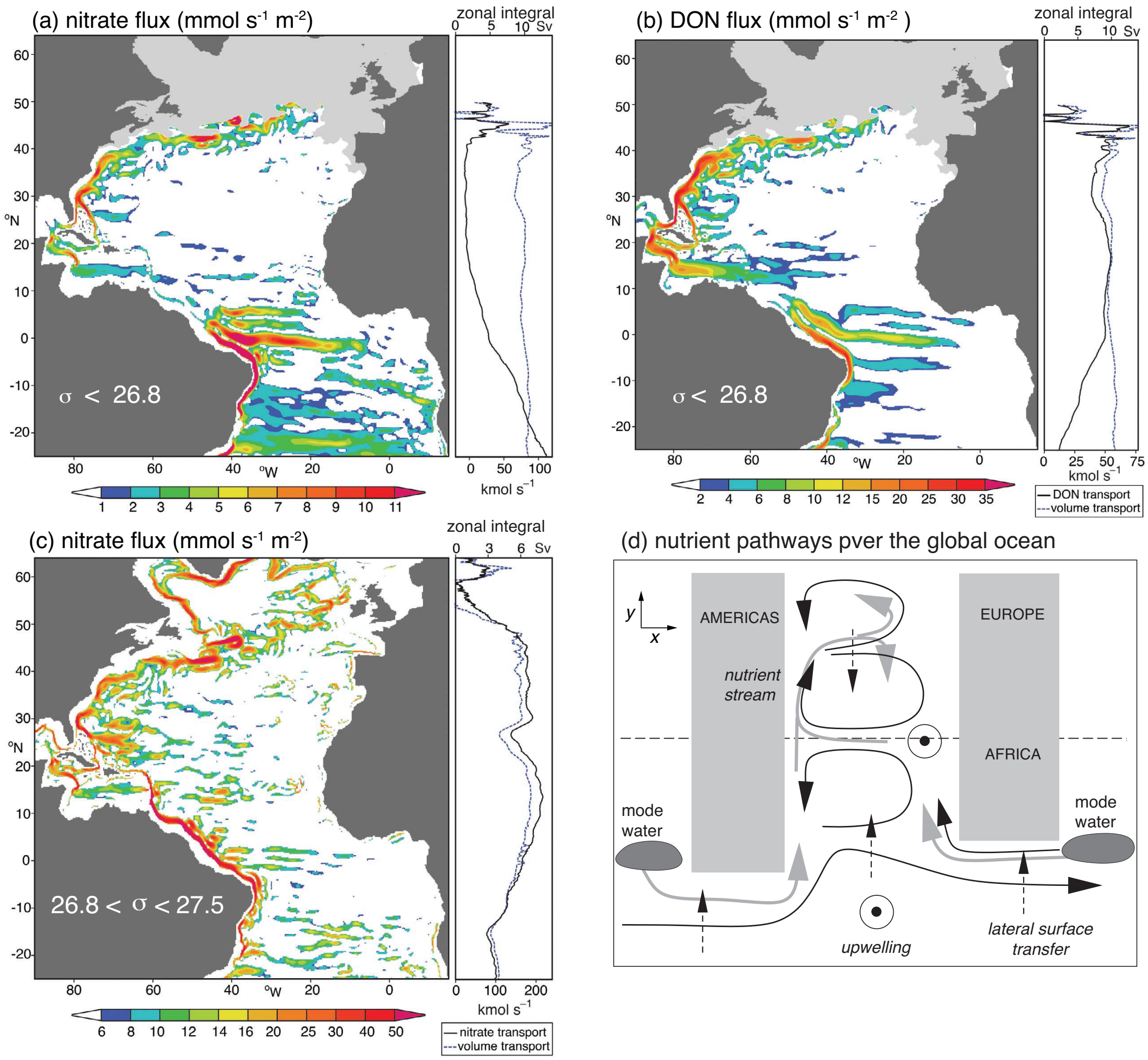

Figure S1. Modelled nutrient stream over the Atlantic. Eddy-permitting model simulations of the nutrient stream[1] (as in Fig. 3): (a) the horizontal nitrate flux (mmol $s^{-1}m^{-2}$) integrated over density layers less than $\sigma = 26.8$ kg m^{-3}; (b) the horizontal dissolved organic nitrogen flux (mmol $s^{-1}m^{-2}$) for the same layers as in (a); and (c) the horizontal nitrate flux (mmol $s^{-1}m^{-2}$) for density layers integrated over $26.8 < \sigma < 27.5$ kg m^{-3}. In the right side panels, there is the meridional volume transport over the width of the basin for those layers (Sv, blue line) and the associated nutrient transport (kmol s^{-1}, black line). In (d), a schematic view of the nutrient stream[2] (light grey arrows) in the Atlantic. Sub-Antarctic mode waters (dark grey) are formed in the southern hemisphere, transported northward and eventually transferred into the downstream mixed layer; the transport involves the horizontal Ekman (dashed line), gyre (black) and intermediate (grey) circulations.

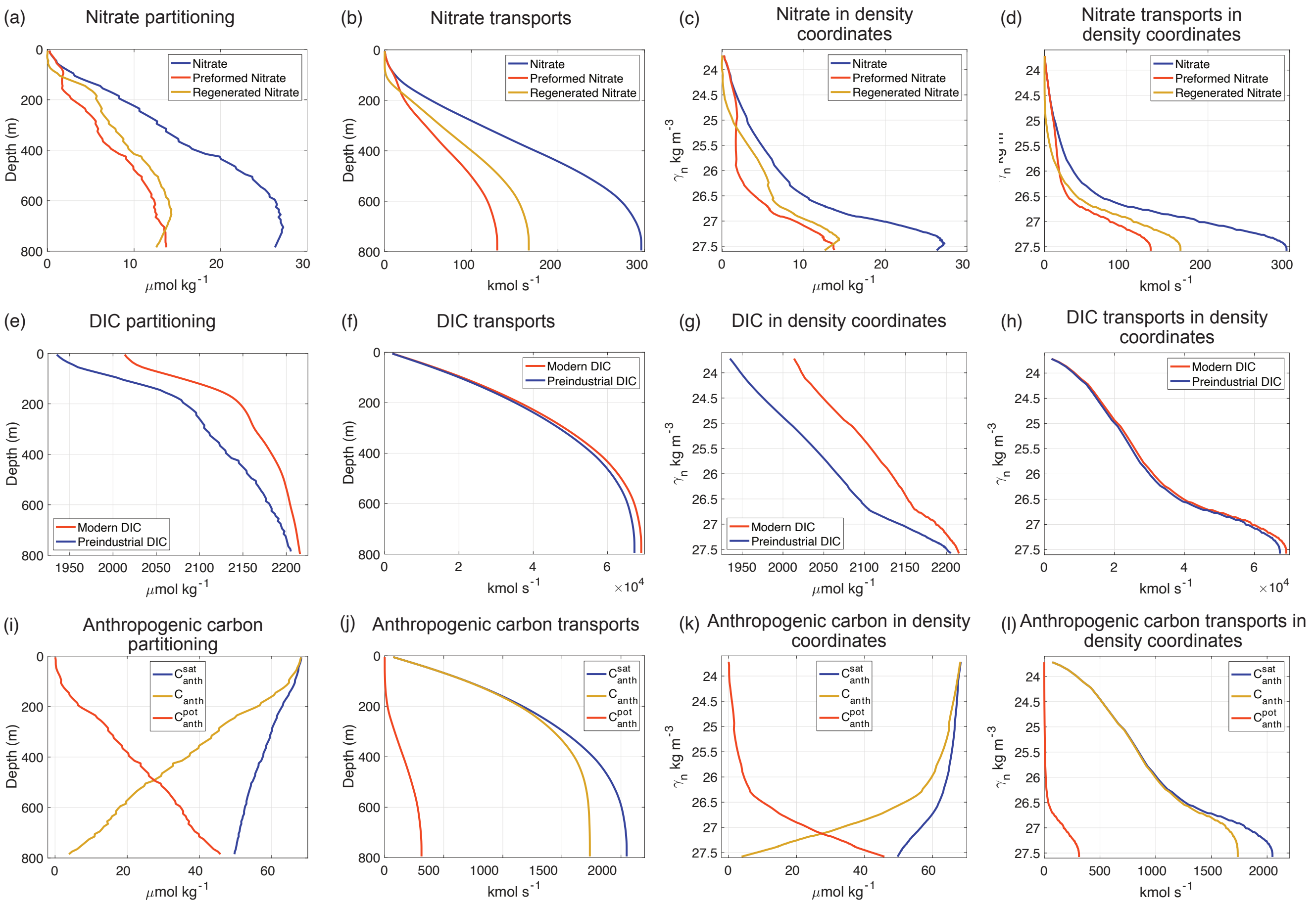

Figure S2. Biogeochemical section and horizontal transports in the Florida Straits. Observation of biogeochemical properties and associated transports at 27°N (as in Fig. 4): (a) nitrate (blue line) partitioning into preformed (orange line) and regenerated (yellow line) nutrients (μmol kg^{-1}); (b) nitrate transport integrated from the surface versus depth and across the Florida Straits (kmol s^{-1}); (c) nitrate in density coordinates (μmol kg^{-1}); and (d) nitrate transport integrated from the surface versus neutral density (kmol s^{-1}); (e) DIC (blue line) and pre-industrial DIC (orange line) (μmol kg^{-1}); (f) DIC transport integrated from the surface versus depth (kmol s^{-1}); (g) DIC versus density (μmol kg^{-1}) and (h) associated transport integrated from the surface versus neutral density (kmol s^{-1}); (i) anthropogenic carbon (blue line) together with saturated anthropogenic carbon (orange line) that is the maximum possible anthropogenic carbon that can be held and potential to uptake additional anthropogenic carbon (yellow line) (μmol kg^{-1}); (j) anthropogenic carbon transport integrated from the surface versus depth (kmol s^{-1}); (k) anthropogenic carbon versus density and (l) associated transport integrated from the surface versus neutral density (kmol s^{-1}).

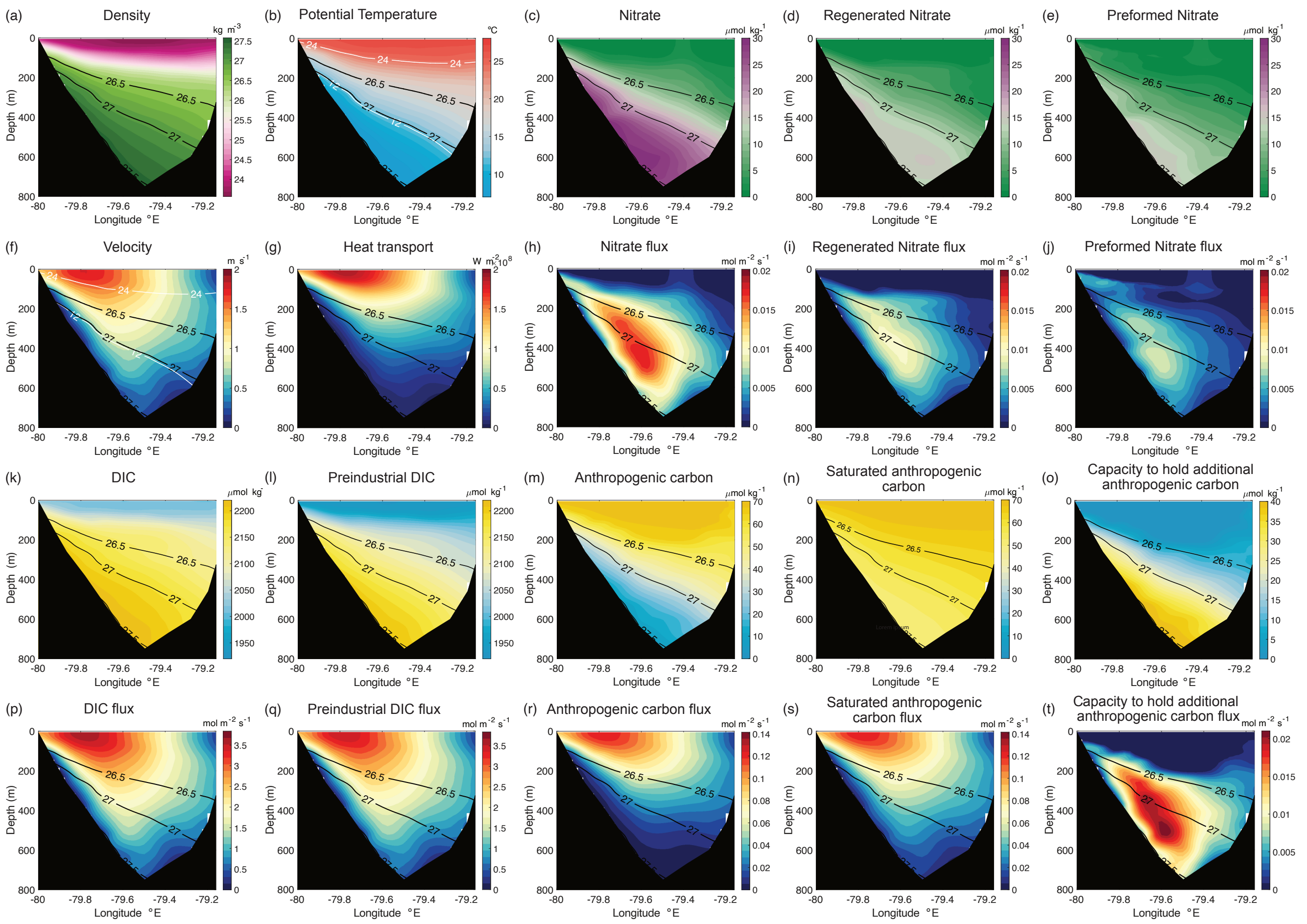

Figure S3. Biogeochemical section and horizontal fluxes in the Florida Straits. Observation of biogeochemical properties and associated horizontal fluxes at 27°N (as in Fig. 4): (a) density - 1000 kg m^{-3}; (b) potential temperature (^{o}C); (c) nitrate (μmol kg^{-1}); (d) regenerated nitrate (μmol kg^{-1}); (e) preformed nitrate (μmol kg^{-1}); (f) velocity (m s^{-1}); (g) northward heat flux (10^{8}W m^{-2}; (h) northward nitrate flux (mol m^{-2}s^{-1}) (from the product of velocity, density and nitrate); (i) northward regenerated nitrate flux (mol m^{-2}s^{-1}); (j) northward preformed nitrate flux (mol m^{-2}s^{-1}); (k) DIC (μmol kg^{-1}); (l) pre-industrial DIC (μmol kg^{-1}); (m) anthropogenic carbon (μmol kg^{-1}); (n) saturated anthropogenic carbon (μmol kg^{-1}); (o) capacity to hold additional anthropogenic carbon (μmol kg^{-1}); (p) DIC northward flux (mol m^{-2}s^{-1}); (q) pre-industrial DIC northward flux (mol m^{-2}s^{-1}); (r) anthropogenic carbon northward flux (mol m^{-2}s^{-1}); (s) saturated anthropogenic carbon northward flux (mol m^{-2}s^{-1}); and (t) capacity to hold additional anthropogenic carbon northward flux (mol m^{-2}s^{-1}).
