# Supplementary material for: The biogeochemical transport by the Gulf Stream
Source: Commun Earth Environ. 2026 Feb 12;7(1):149. doi: 10.1038/s43247-025-03118-y (PMC12900640; doi:10.1038/s43247-025-03118-y)
Supplement: Supplementary file 3 — Description of Additional Supplementary File [file 43247_2025_3118_MOESM3_ESM.pdf]

Description of Additional Supplementary Files

File name: Supplementary Video

Description: Video linked to the Perspective. Also available at https://www.youtube.com/watch?v=dyVdmvvAjkc
